# A comprehensive catalogue of plant-pollinator interactions for Chile

**DOI:** 10.1038/s41597-022-01195-8

**Published:** 2022-03-11

**Authors:** Giselle Muschett, Francisco E. Fontúrbel

**Affiliations:** Instituto de Biología, Pontificia Universidad Católica de Valparaíso, Av. Universidad 330, Valparaíso, 2373223 Chile

**Keywords:** Entomology, Biodiversity, Conservation biology

## Abstract

Pollinators and pollination services provide invaluable ecosystem services to agriculture and contribute to the maintenance of biodiversity. In Chile, pollination contributes greatly to the diversity of native ecosystems and provides ecosystem services to crops, but local pollinator abundance and diversity, as well as plant-animal interactions, remain poorly understood. We compiled all available information from the published scientific literature on pollinators, flower visitors, and plant-pollinator interactions in Chile and found 120 publications from which we extracted 2619 records. Those records contain locality, habit type, and establishment means of 357 plant species from 83 families. Thus, we built a database compiling information on their pollinators and flower visitors, including information on 492 pollinator species from 97 families and 13 orders. Our database provides the first systematisation of information about pollinators and pollination in Chile. This country relies heavily on pollinators both for its agricultural industry and its unique and highly endemic biodiversity. This information can be reused in future studies and would contribute significantly to pollinator conservation strategies.

## Abstract (summary)

**Table:** 

Measurement(s)	pollination
Technology Type(s)	Observation
Factor Type(s)	Pollination
Sample Characteristic - Organism	Aves • Insecta • Mammalia • Magnoliophyta
Sample Characteristic - Environment	habitat
Sample Characteristic - Location	Chile

## Background & Summary

In recent years there has been an increasing concern regarding the global decline of pollinators and pollination services^1–3^. Recent studies estimate that over 87% of the flowering plant species rely on biotic pollination^4^. Pollination is a mutualistic interaction, and plants provide pollinators with various rewards, including nectar, oil, or excess pollen to feed upon^5,6^. Although bees are the most well-known pollinator group, pollination can be performed by a wide variety of species, including mammals, birds, reptiles, and other insects.

Plant-pollinator interactions are among the key processes that generate and maintain biodiversity^7,8^. The coevolutionary processes involved in animal pollination have helped maintain the structure and function of entire communities and species’ networks. Wild plant species and natural ecosystems provide several products and services, including nutrient cycling, medicine, food, a source of pollinators for domesticated crops, and alternative food and shelter sources for agricultural pollinators^9^. However, the complex web of interactions and the large number of species involved (ca. 400,000 species globally) makes it challenging to estimate pollinators’ value in natural ecosystems, particularly when the life history of so many pollinator species remains little studied and understood^10^.

Pollinators also provide highly valuable ecosystem services to crops^11,12^. More than 70% of the world’s crops depend directly on insect pollination, making pollination key to food security^11,13^. The European honeybee (*Apis mellifera*) is likely the most economically important pollinator of crops worldwide^13,14^. Honeybees are adaptable, easy to manage, and cost-efficient. However, in recent years, ‘colony collapse’ caused by several factors, including parasitic mites and the excessive use of pesticides and herbicides, have led to a decline in managed honeybee colonies in many parts of the world^15–17^. Similarly, habitat loss and fragmentation have detrimental effects on both native and commercial pollinators. In degraded habitats, pollinators struggle to find resources and nesting sites^18–20^.

In Chile, pollination represents a multimillion-dollar business. Between January and October 2020, the export of Chilean fruit reached USD 4.149 million, while fresh vegetables generated USD 347 million during the same period^21^. Although agricultural pollinators have been well studied, native pollinators remain largely unknown. With over 460 species of native bees in Chile, approximately 70% are endemic; researchers have only begun to understand the relationships between native plants and their pollinators^22–24^. Also, managed honeybees and bumblebees introduced to Chile for crop pollination are highly invasive and easily leave croplands to forage in neighbouring native ecosystems^25,26^, competing directly with native pollinators for the ever-diminishing resources in native grasslands and forests posing a threat to Chile’s unique ecoregions^25,27^.

Because of the importance of pollination in the maintenance of biodiversity and the economic benefits of agricultural crop production, there is an urgent need to understand the causes behind the current decline in pollinator species. In this sense, collating and reviewing existing information on pollinators and making this information easily accessible in the form of a user-friendly database is of immeasurable value. In this study, we compiled the information available about pollination and pollinators (*sensu lato*) for Chile, aiming to understand plant-pollinator interactions, identify knowledge and geographic gaps, and provide a baseline from which to carry out further studies. We aimed to make a datasheet with a format that was adaptable to different regions and other countries by allowing it to be easily understood, easy to access and find and aiming to avoid duplicity of data. This study represents the first systematic effort to compile the available information on pollination and pollinators for Chile. This pollination catalogue for Chile adds to other international efforts of systematising this information as, for example, the Catalogue of Afrotropical Bees^28^ and the CPC Plant Pollinators Database^29^.

## Methods

We carried out a systematic compilation of published scientific literature on pollinators, flower visitors, and plant-pollinator interactions of Chile obtained from Web of Science. We used the following search terms: ‘Chile’ and ‘pollinat*’ or ‘flower*’. Also, we consulted studies referenced in these articles that were not included in Web of Science. Many of these referenced studies not included in the Web of Science were articles published in local journals, government reports, and other grey literature. We selected those that specifically mentioned study areas within Chile and studies that listed geographic coordinates that corresponded to Chile from this pool of studies. From this second set of studies, we assessed the information on pollinators and plant-pollinator interactions. We considered plant-pollinator interactions to include visitation rates, plant reproductive systems, the capture of pollinators to assess/collect pollen grains, and those studies where the authors defined pollination in the Methods section of the manuscript as an animal coming in contact with the plant reproductive structures. Otherwise, we listed those interactions as flower visitation events (all pollinators are flower visitors, but not all flower visitors are pollinators).

How plant-pollinator data is collected and the type of data collected limit the ability to share and collate the information. To simplify how we assessed the information in these studies, aimed at creating a unified and integrated data collection, we used the DarwinCore standard^30^. DarwinCore standard provides a way to systematise heterogeneous sources of data on biodiversity, intending to improve interoperability. The standard simplifies data collection and analysis by defining categories of common terms, creating a standard language or controlled vocabulary, providing researchers with a unifying format that facilitates the use of the information in future analyses. We chose a subset of 38 DarwinCore terms to classify our data from the following classes: Record Level, Occurrence, Organism, Event, Location, Identification, Taxon, and Measurement. As we have occurrence data for both animal and plant species in the same database, we created some terms based on DarwinCore, for example we have ‘scientificNamePlants’ and ‘scientificNameAnimals’ based on ‘scientificName’ to differentiate them. However, DarwinCore was not designed to manage information that has already been published with differing degrees of specificity regarding the field methodology used or to handle plant-pollinator interactions. Thus, it lacks terms that allow the description of publications and interactions. To address this challenge, we used terms from the TDWG’s Plant-Pollinator Interaction (PPI; https://www.tdwg.org/community/interaction/) Standard. The PPI is still in its development stages but was designed for the Safeguarding Pollination Services in a Changing World project (SURPASS2) to standardise and manage plant-pollinator interactions’ complexities. To that end, it has terms that detail the types of interactions between flowers and animals (e.g., pollinates, visits, nectar rob, feeds). From the PPI we used four terms: Self Incompatibility, Habit, and Resource Collected. Information regarding the Interaction type was based on the Ontobee Relationship Ontology nomenclature (http://www.ontobee.org/ontology/RO). We also used the Ecological Metadata Language (EML; https://eml.ecoinformatics.org) to include fields related to the publication date and coordinates that bound the study area when no specific coordinates were stated in the publication. We used ISO 8601 format to report event dates or date ranges. A summary of the terms included in this database and its sources is presented in Table 1.

**Table 1:** Terms used in the database and their sources.

Character	Source
datasetID	DwC
creator	EML
eventDate	ISO8601
pubDate	EML
reference	DwC
country	DwC
stateProvince	DwC
municipality	DwC
verbatimSite	DwC
decimalLatitude	DwC
decimalLongitude	DwC
northBoundingCoordinate	EML
southBoundingCoordinate	EML
verbatimCoordinates	DwC
minimumElevationInMeters	DwC
maxElevationInMeters	DwC
verbatimElevation	DwC
organismQuantityPlants	Based on DwC
identificationQualifierPlants	Based on DwC
scientificNamePlants	Based on DwC
classPlants	Based on DwC
familyPlants	Based on DwC
genusPlants	Based on DwC
specificEpithetPlants	Based on DwC
infraspecificEpithetPlants	Based on DwC
taxonRankPlants	Based on DwC
taxonRemarksPlants	Based on DwC
establishmentMeansPlants	Based on DwC
habitPlants	PPI
selfIncompatabilityPlants	PPI
organismQuantityAnimals	Based on DwC
identificationQualifierAnimals	Based on DwC
scientificNameAnimals	Based on DwC
classAnimals	Based on DwC
orderAnimals	Based on DwC
familyAnimals	Based on DwC
subfamilyAnimals	Based on DwC
genusAnimals	Based on DwC
specificEpithetAnimals	Based on DwC
taxonRankAnimals	Based on DwC
taxonRemarksAnimals	Based on DwC
establishmentMeansAnimals	Based on DwC
interactionType	Based on Ontobee
interactionTypeIRI	Ontobee
resourceCollected	PPI
samplingProtocol	DwC
sampleSizeValue	DwC
samplingSizeUnit	DwC

Those terms listed as “Based on” indicate that we made modifications to the original term.

From the publications obtained, we extracted locality information, geographic coordinates and elevation (when available), the date and duration of the study, plant species name or genus, family, degree of self-compatibility, habit, and establishment means. Then, for each corresponding site and plant species, we compiled information on their pollinators and flower visitors, including the species and genus, family, and order of the animal, the type of interaction (specified by the authors as a pollinator, visitor, or nectar robber), type of resource collected (e.g., nectar or pollen), the methodology used (e.g., focal observations), the sampling unit and duration (e.g., 30 minutes). In addition to the characteristics mentioned above, we also included the full reference of the source material. Table S1 (available online as Supplementary Material) lists these characteristics and provides a detailed description of each variable.

## Data Records

**Fig. 1:**
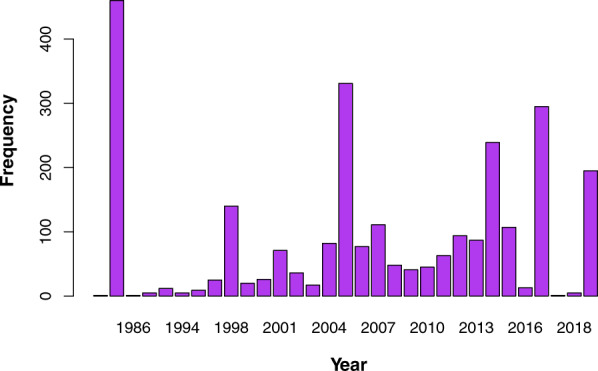
Temporal coverage of the studies on pollination included in the dataset.

**Fig. 2:**
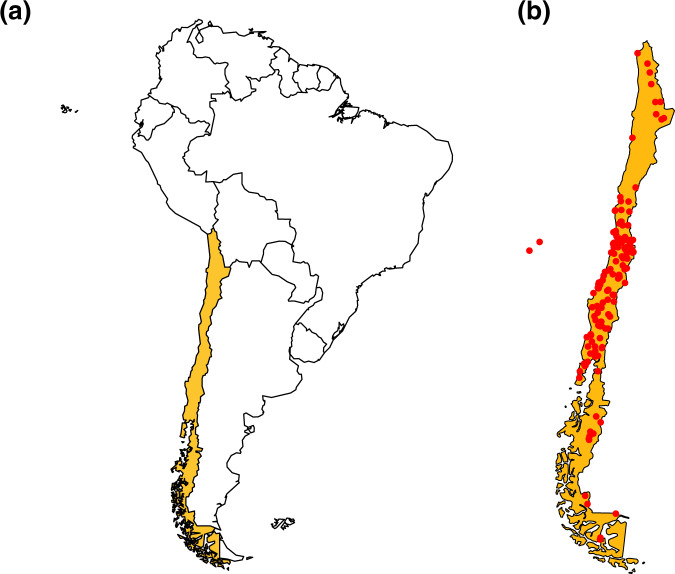
Location of (**a**) Chile within South America and (**b**) sampling localities of the pollination studies included in Chile’s dataset.

**Fig. 3:**
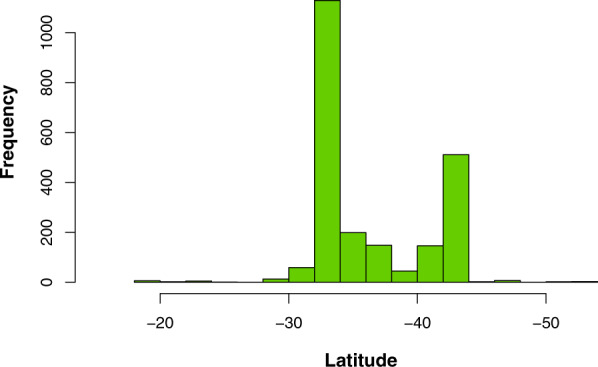
Latitudinal range of the studies on pollination included in the dataset.

We generated a database with 120 publications that span between 1982 and 2019 (Fig. 1). From these publications, we extracted 2619 records containing information on locality, geographic coordinates and elevation. The records represent 357 plant species belonging to 83 families, degree of self-compatibility, habit type (e.g. tree, bush), and plant establishment means (i.e., native or introduced). With those records, we built a large database compiling information on their pollinators and flower visitors, which yielded information on 492 species belonging to 97 families and 13 orders, type of interaction as specified by the authors, type of resource collected, the methodology used, the sampling unit, and duration/number of sampling units. The majority of records (48%) correspond to bees, though other insects are also well represented (41%), while vertebrates (mainly hummingbirds) make up only 11% of the total records. Besides, 86% of records have associated geographic coordinates and cover most of the long Chilean continental territory (Fig. 2), from 18.5° to 53.7°S (Fig. 3), and includes records from the Juan Fernández Islands. All records are available from the figshare digital repository (10.6084/m9.figshare.14743881)^31^.

## Technical Validation

The data presented in this study adheres to the FAIR principles: Findability, Accessibility, Interoperability, and Reuse of digital assets.

Our initial search yielded 175 publications, but after assessing the data for a series of particular characteristics, including coordinates and specific plant-pollinator interactions, we obtained a final set of 120 publications. From this second set of publications, we extracted the specific parameters mentioned in Table S1. The dataset was then checked for grammar and spelling and the taxonomic status of identified species. In several instances, scientific names had changed since the article was published, or taxonomic relationships had changed, or the scientific name in the original publication was misspelt. We used OpenRefine 3.4 software to curate and validate data as well as the Global Biodiversity Information Facility (GBIF; https://www.gbif.org) species backbone to validate scientific names and taxonomy. Finally, to ensure accurate taxonomic relationships, the list of unidentified or questionable species was sent to an independent taxonomist for review. In these cases, we listed the correct or updated scientific name and the original scientific name was reported in the “taxonomicRemarks” column. There were five species (*Alstroemeria exerens*, *Eurymetopum obscurum*, *Eurymetopum prasinum*, *Eurymetopum proteus*, and *Yramea modesta*) not listed under GBIF species backbone, but they are valid species for Chile.

We verified the geographic coordinates obtained using Google Earth version 7.3 to ensure all locations were indeed within Chile and corresponded to the verbatim site as reported in the article or publication. All sites complied with the parameters established. Besides, the plant-animal interaction was carefully scrutinised. An animal was only considered to pollinate a plant if the authors mentioned the animal making contact with the plant’s reproductive structures. If not, the interaction was labelled as “flower visitor”, and no resource was reported to be collected. Similarly, each plant species and its corresponding interaction with an animal species represented one record. For example, there are records where there are only two plant species reported in the publication, but 15 animal species that pollinated/visited these two species. This record will have 30 data rows due to the careful methodology applied, one for each animal-plant species combination.

## Usage Notes

Our database contains records that can be analysed using R or other languages for data processing. Our dataset can be easily combined with similar data using a simple R script (provided in the GitHub repository) and standard tools that read DarwinCore fields (e.g., the *rgbif* package in R).

As this database provides a baseline for Chile regarding pollination and pollinators (*sensu lato*), it could be reused to generate data papers as a data source for new research (e.g., interaction networks) to inform decision-making processes. As most of the data points presented contain geographic coordinates, this information will enable users to plot plant and pollinator locations in a way and at a scale that has not been possible before in Chile, allowing users to conduct spatially-explicit analyses if necessary. This type of data will allow further analyses such as studying local or regional pollinator declines, pollinators’ behavioural ecology, and the reproductive biology of many native and agricultural plant species. Many of the studies included in the database also provide detailed information on native plants’ flowering periods, flower abundance, and local abundance of pollinators, allowing for more complex analyses of these characteristics across the entire country. We believe the database provided here will be of unique importance to the study of pollinators in Chile.

## Supplementary information

Table S1

## Data Availability

The supporting data associated with this article (provided in. csv format), the list of articles proving data (presented in.ris and.bib formats), and the code used (in R language) to describe the data and generate maps from it are available from GitHub: https://github.com/fonturbel-lab/pollination_catalogue (10.5281/zenodo.4445125)^32^. A mirror repository is available from figshare (10.6084/m9.figshare.14743881)^31^, which is automatically updated from GitHub. All information is provided under a Creative Commons (CC0) license.
