# Supplementary material for: A comprehensive catalogue of plant-pollinator interactions for Chile
Source: Sci Data. 2022 Mar 11;9:78. doi: 10.1038/s41597-022-01195-8 (PMC8917211; doi:10.1038/s41597-022-01195-8)
Supplement: Supplementary file 1 — Table S1 [file 41597_2022_1195_MOESM1_ESM.pdf]

SUPPLEMENTARY MATERIAL

A comprehensive catalogue of plant-pollinator interactions for Chile

Giselle Muschett & Francisco E. Fontúrbel

Table S1. Description of the terms used to assess the publications reviewed in this study.

Character	Description
datasetID	A unique identifier for the set of data, which may be an identifier specific to a collection or institution
creator	Name(s) of people, groups, or organisations responsible for recording the original Occurrence
eventDate	The date-time or interval during which an event occurred. We used the format YYYY-MM-DD to express year, month, and day (when available). Date ranges were separated by a slash (/) mark
pubDate	Date bibliographic citation was published
reference	A related resource that is referenced, cited, or otherwise pointed to by the described resource
country	Name of the country or major administrative unit in which the Location occurs
stateProvince	The name of the next smaller administrative region than country (state, province, region) in which the Location occurs
municipality	The full, unabbreviated name of the next smaller administrative region than county (city, municipality, etc.) in which the Location occurs. Do not use this term for a nearby named place that does not contain the actual location
verbatimSite	The original textual description of the place
decimalLatitude	The geographic latitude (in decimal degrees, using the spatial reference system given in geodeticDatum) of the geographic center of a Location. Positive values are north of the Equator, negative values are south of it

Character	Description
decimalLongitude	The geographic longitude (in decimal degrees, using the spatial reference system given in geodeticDatum) of the geographic center of a Location. Positive values are east of the Greenwich Meridian, negative values are west of it
northBoundingCoordinate	Latitude of the northern-most point of the area that is being described
southBoundingCoordinate	Latitude of the southern-most point of the area that is being described
verbatimCoordinates	The verbatim original spatial coordinates of the Location
minimumElevationInMeters	The original description of the elevation (altitude above sea level) of the Location
maxElevationInMeters	The upper limit of the range of elevation (altitude above sea level), in meters
verbatimElevation	The original description of the elevation (altitude above sea level) of the Location
organismQuantity Plants	Quantity of organisms, in this case plant species cited in the study
scientificName Plants	The full scientific name. This should be the name in lowest level taxonomic rank that can be determined
classPlants	The full scientific name of the class in which the taxon is classified
familyPlants	The full scientific name of the family in which the taxon is classified
genusPlants	The full scientific name of the genus in which the taxon is classified
specificEpithetPlants	The name of the first or species epithet of the scientificName
infraspecificEpithetPlants	The name of the lowest or terminal infraspecific epithet of the scientificName, excluding any rank designation
taxonRankPlants	The taxonomic rank of the most specific name in the scientificName
taxonRemarksPlants	Original spelling of scientific name, genus or taxonomic relationship of plant species reported in the source paper
establishmentMeansPlants	Statement about whether an organism or organisms have been introduced to a given place and time through the direct or indirect activity of modern human
habitPlants	The general appearance, characteristic form, or mode of growth of a plant species represented in the record, such as tree, bush, climber.

Character	Description
selfIncompatabilityPlants	The type or class of the self-incompatibility system of the plant species
organismQuantityAnimals	Quantity of organisms, in this case animal species cited in the study
scientificNameAnimals	The full scientific name. This should be the name in lowest level taxonomic rank that can be determined
orderAnimals	The full scientific name of the order in which the taxon is classified
classAnimalss	The full scientific name of the class in which the taxon is classified
familyAnimals	The full scientific name of the family in which the taxon is classified
genusAnimals	The full scientific name of the genus in which the taxon is classified
genusAnimals	The full scientific name of the genus in which the taxon is classified
specificEpithetAnimals	The name of the first or species epithet of the scientificName
infraspecificEpithetAnimals	The name of the lowest or terminal infraspecific epithet of the scientificName, excluding any rank designation
taxonRankAnimals	The taxonomic rank of the most specific name in the scientificName
TaxonRemarksAnimals	Original spelling of scientific name, genus or taxonomic relationship of plant species reported in the source paper
establishmentMeansAnimals	Statement about whether an organism or organisms have been introduced to a given place and time through the direct or indirect activity of modern human
interactionType	Statement about the type of interaction between plant and animal
interactionTypeIRI	Ontobee URL resource associated to interactionType
resourceCollected	The floral resource used or removed by an animal
samplingProtocol	The method or protocol used during an Event
sampleSizeValue	A sample Size Unit must have a corresponding sample Size Value. Recommended best practice is to use a controlled vocabulary such as the Ontology of Units of Measure of SI units

Character	Description
samplingSizeUnit	The unit of measurement of the size (time duration, length, area, or volume) of a sample in a sampling event
